# CFTR dysfunction leads to defective bacterial eradication on cystic fibrosis airways

**DOI:** 10.3389/fphys.2024.1385661

**Published:** 2024-04-18

**Authors:** Min Wu, Jeng-Haur Chen

**Affiliations:** College of Life Sciences, Zhejiang Normal University, Jinhua, Zhejiang, China

**Keywords:** cystic fibrosis, CFTR, bacterial infection, airway epithelia, mucus, bicarbonate

## Abstract

Dysfunction of the cystic fibrosis transmembrane conductance regulator (CFTR) anion channel by genetic mutations causes the inherited disease cystic fibrosis (CF). CF lung disease that involves multiple disorders of epithelial function likely results from loss of CFTR function as an anion channel conducting chloride and bicarbonate ions and its function as a cellular regulator modulating the activity of membrane and cytosol proteins. In the absence of CFTR activity, abundant mucus accumulation, bacterial infection and inflammation characterize CF airways, in which inflammation-associated tissue remodeling and damage gradually destroys the lung. Deciphering the link between CFTR dysfunction and bacterial infection in CF airways may reveal the pathogenesis of CF lung disease and guide the development of new treatments. Research efforts towards this goal, including high salt, low volume, airway surface liquid acidosis and abnormal mucus hypotheses are critically reviewed.

## 1 Introduction

Cystic fibrosis (CF) is an inherited autosomal recessive disease caused by mutations in the gene encoding the cystic fibrosis transmembrane conductance regulator (CFTR) (Riordan et al., 1989), an epithelial anion channel that primarily conducts Cl^−^ and HCO_3_^−^ (Anderson et al., 1991; Poulsen et al., 1994). CF mutations impair CFTR function mainly by severely disrupting protein expression and channel function in the cell membrane (Amaral and Farinha, 2013), leading to abnormal water absorption (Matsui et al., 1998), mucus secretion (Joo et al., 2002), pH regulation (Pezzulo et al., 2012), bacterial infection (Reece et al., 2021) and inflammation (Khan et al., 1995) in many epithelia-lined organs, including the lungs, intestine, liver, pancreas, sweat glands and reproductive tract (Shteinberg et al., 2021). Thus, CF is well known as a multiple-organ disease (Shteinberg et al., 2021).

Nowadays, more than 2000 CFTR mutations have been identified (Shteinberg et al., 2021), and at least 719 mutations that cause CF are reported (https://cftr2.org/) (Sosnay et al., 2013). Currently, lung failure due to chronic and recurrent bacterial infection is the major cause of death in people with CF (Ciofu et al., 2013). Bacteria *Staphylococcus aureus* (Durfey et al., 2021) and *Haemophilus influenza* (Green and Jones, 2022) are commonly observed in the airways of both young and adult people with CF. Moreover, *Pseudomonas aeruginosa* is the bacteria particularly noted and observed in CF airways with chronic lung infections (Lund-Palau et al., 2016; Durfey et al., 2021). Therefore, understanding how CFTR dysfunction impairs the defense mechanisms of airway epithelia against bacterial infection is critical for exploring an effective treatment for CF.

CFTR involves two major physiological functions in epithelial cells. First, CFTR is well characterized as a Cl^−^ channel (Anderson et al., 1991); it also partially conducts HCO_3_^−^ (Poulsen et al., 1994), but little Na^+^ (Tabcharani et al., 1997). In CF, the patient who carries mutant CFTR producing the least amount of cAMP-stimulated Cl^−^ current displays the worst exocrine pancreatic deficiency (Sheppard et al., 1993; Sheppard et al., 1995), suggesting that defective Cl^−^ transport would be a possible cause of CF disease. In CF epithelia, lack of CFTR-mediated Cl^−^ conductance may hinder the apical Na^+^ absorption, so that NaCl may be retained in the airway surface liquid (ASL) resulting in the “high salt” condition, which impairs bacterial killing in ASL (Smith et al., 1996). In addition, the lack of HCO_3_^−^ transport in genetically modified *CFTR* ^*−/−*^ newborn pigs decreases ASL pH resulting in reduced bacterial killing upon airway epithelia, suggesting that “ASL acidosis” is a major defect leading to CF lung disease (Pezzulo et al., 2012).

It is of interest that CFTR proteins are abundantly expressed in forkhead box I1 (FOXI1)-positive pulmonary ionocytes (Montoro et al., 2018; Plasschaert et al., 2018), while airway epithelia of FOXI1-knockout mice display increased ciliary beat frequency and mucus viscosity (Montoro et al., 2018). Lei et al. (Lei et al., 2023) demonstrated that apical membrane CFTR Cl^−^ channels collaborate with basolateral membrane barttin/ClC-K Cl^−^ channels in ionocytes for transepithelial Cl^−^ absorption, leading to fluid absorption. Moreover, FOXI1-knockout and CF ferrets both display reduced ASL volume and impaired mucociliary clearance due to ASL abnormalities, including slow fluid absorption but also absent fluid secretion, lack of CFTR-mediated ASL alkalization and increased mucus viscosity (Yuan et al., 2023). Therefore, albeit less than about 1% of total epithelial cells in airways (Montoro et al., 2018; Plasschaert et al., 2018; Lei et al., 2023), ionocytes regulate ASL homeostasis. However, whether the dysfunction of ionocytes contributes to the pathogenesis of CF lung diseases needs to be further explored.

Second, CFTR regulates the activity of several membrane and cytosol proteins (Li and Naren, 2010; Lim et al., 2017). Normally, CFTR-mediated transepithelial Cl^−^ transport together with the epithelial Na^+^ channel (ENaC)-mediated Na^+^ transport control the salt and water absorption of epithelia (Matalon et al., 2015). Early studies (Kunzelmann et al., 1995; Stutts et al., 1995; Ismailov et al., 1996; Matsui et al., 1998; Tarran et al., 2005) proposed a popular mechanism by which the loss of CFTR function in CF epithelia might enhance ENaC activity, resulting in excess NaCl and water absorption, followed by reduced ASL height that impairs both the mucociliary clearance mechanism and bacterial eradication from the airway surface. In addition to this “low volume” hypothesis, CFTR acting as a regulator also interacts with and stimulates the Cl^−^/HCO_3_^−^ exchanger (Lee et al., 1999), but inhibits Na^+^/H^+^ exchanger (NHE) (Ahn et al., 2001). Moreover, CFTR directly binds to many intracellular proteins via its regulatory (R) domain (Bozoky et al., 2013) and N- (Naren et al., 1997) and C-termini (Hall et al., 1998; Li and Naren, 2010). Thus, CF mutations may disrupt CFTR function as an anion channel and regulator in epithelial cells. However, which defective CFTR function primarily leads to CF lung diseases remains in debate.

A strategy to address this question is to explore which type of CFTR function, once impaired, causes recurrent airway bacterial infection, a key step that elicits excess inflammatory responses and consequent tissue damage in the CF lung (Cabrini et al., 2020; Ribeiro et al., 2023). The physicochemical properties and movement of mucus is another key factor altered upon CF epithelia (Matsui et al., 1998; Hoegger et al., 2014; Esther et al., 2019; Keith et al., 2022). It is noted that accumulated and abnormal mucus disrupts the mucociliary clearance mechanism and impairs bacterial eradication from the airway surface (Matsui et al., 1998; Boucher, 2007). Moreover, bacterial killing on the apical side of epithelia is attenuated in CF (Smith et al., 1996; Pezzulo et al., 2012). Therefore, both mucus accumulation and weakened bactericidal activity in ASL may prevent CF airway epithelia from removing airborne bacteria and lead to bacterial colonization (Stoltz et al., 2015; Ribeiro et al., 2023).

However, the link between CFTR dysfunction and bacterial colonization in CF airways remains incompletely understood. This review will first describe the improvement of CF lung function by the restoration of CFTR activity in the epithelial cells, secondly discuss the epithelial defense mechanisms that remove bacteria from the surface of airways, and then update current understanding of how CFTR dysfunction disrupts these defense mechanisms, leading to bacterial colonization on CF epithelia.

## 2 The cocktail drug therapy of CFTR potentiators and correctors

One way to understand how CFTR dysfunction causes loss of bacterial eradication from the airways is to examine the improvement in lung function when CFTR function is restored pharmaceutically by potentiators that enhance its channel activity and correctors that increase CFTR protein expression at the plasma membrane (Ribeiro et al., 2023; Thornton and Parkins, 2023). Recently FDA-approved triple therapy with elexacaftor/tezacaftor/ivacaftor (ETI) (Keating et al., 2018; Heijerman et al., 2019; Middleton et al., 2019) has been used to treat people with cystic fibrosis carrying at least one ΔF508 allele (Tummler, 2023), in which ivacaftor (VX-770) acts as a potentiator to enhance the channel activity of the ΔF508 or G551D mutants (Van Goor et al., 2009); tezacaftor (VX-661) (Rowe et al., 2017; Taylor-Cousar et al., 2017) and elexacaftor (VX-445) (Keating et al., 2018; Laselva et al., 2021) work as correctors but elexacaftor also partially acts as a potentiator (Laselva et al., 2021). The promising outcomes show that the combination treatment substantially recovers CF lung function by reducing pulmonary exacerbations and hospitalizations per patient per year (Bower et al., 2023), improving percent predicted forced expiratory volume in 1 s (ppFEV1) up to 10% (Bower et al., 2023; Atteih et al., 2024), diminishing the release of inflammatory mediators, such as the B cell activating factors, IL-6, IL-8 and IL-22, C-reactive protein and soluble TNF (Casey et al., 2023; Schaupp et al., 2023; Atteih et al., 2024), advancing sputum viscoelastic properties (Schaupp et al., 2023), lowering the sweat Cl^−^ concentration and lung clearance index_2.5_ (Goralski et al., 2023), and decreasing the detection and diversity of bacteria like *S. aureus* and *P. aeruginosa* in CF microbiological samples (Dittrich et al., 2024) and sputum (Nichols et al., 2023; Schaupp et al., 2023). Notably, ETI treatment is safe for use in children aged 2–5 years (Goralski et al., 2023) and pregnant women (Cimino et al., 2024).

However, ETI only shows moderate increases in ppFEV1 in subjects who were ineligible for enrollment in registration studies and those with severe airway obstruction (ppFEV1 < 40) (Fila et al., 2023). Although ETI treatment reduces positive bacterial cultures in patients (Bower et al., 2023), bacteria not in the CF pathogen genera persist in the sputum of patients and are not changed by ETI treatment (Martin et al., 2023; Nichols et al., 2023). These findings may reflect the complex responses of microbiota to ETI treatment. Details about this topic can be found in previous excellent reviews (Ribeiro et al., 2023; Thornton and Parkins, 2023).

## 3 Bacterial eradication by airway epithelia

**FIGURE 1:**
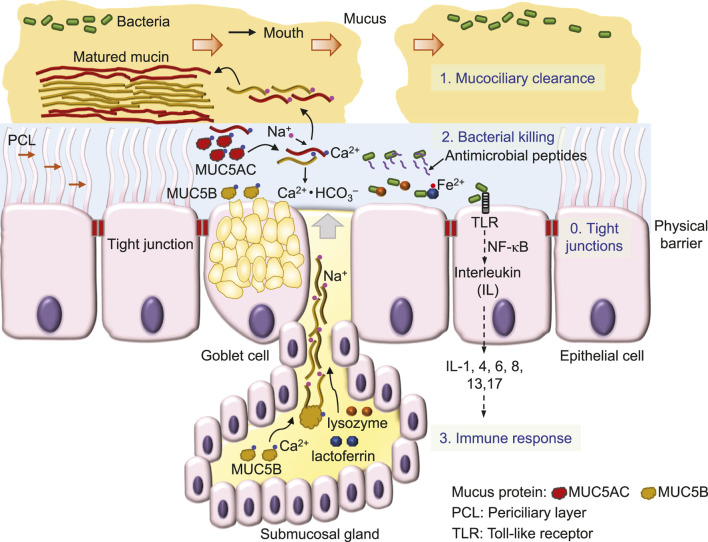
Defense mechanisms of airway epithelia against invading bacteria.

Inhaled air carries various particles, including debris, allergens and pathogens like viruses and bacteria (Costantini et al., 2022). To effectively remove inhaled substances, airway epithelia employ a 4-level defense scheme, known as the innate immune system (Myszor and Gudmundsson, 2023) (Figure 1). A single layer of epithelial cells, connected by tight junctions that firmly seal the apical side of cell membranes, preventing water and ions from passing through the paracellular pathway between cells, forms a physical barrier to serve as the fundamental defense mechanism of airway epithelia against bacteria invasion (Otani and Furuse, 2020; Myszor and Gudmundsson, 2023) (Figure 1). Additional defense mechanisms include 1) the mucociliary clearance mechanism that mechanically removes bacteria from the airway surface (Bustamante-Marin and Ostrowski, 2017), 2) bacterial killing by antimicrobial substances (Geitani et al., 2020) and 3) epithelial inflammatory responses (Yu and Kotsimbos, 2023).

First, the mucociliary clearance mechanism is constructed by viscoelastic and discontinued mucus (Rogers et al., 2022; Abrami et al., 2024) that flow on a watery and gel-like layer, the periciliary layer (PCL) (Widdicombe and Widdicombe, 1995; Button et al., 2012) (Figure 1). Sticky mucus traps inhaled bacteria, debris and other particles, while the cilia of epithelial cells in the underlying PCL propel the mucus and trapped substances (Figure 1) towards to the pharynx, where they form the sputum for expectoration (Bustamante-Marin and Ostrowski, 2017; Rogers et al., 2022).

The major component of this mucociliary clearance mechanism is the mucin protein, an O-linked glycoprotein containing a core protein of MUC5AC (5654 amino acids) secreted from Goblet cells or MUC5B (5762 amino acids) secreted mainly from submucosal glands (Hovenberg et al., 1996; Ermund et al., 2018; Thornton et al., 2018). By disulfide bonds formed between individual proteins, mucins are polymerized into a high-molecular weight glycoprotein of ∼2–50 mD (Thornton et al., 2018).

This large mucin molecule is stored in the vesicles of the cell, secreted upon cell stimulation by various signals and transformed from a granular shape into a flat and extended bundle during the journey to the surface of ASL (Ermund et al., 2018; Jaramillo et al., 2018) (Figure 1). Because of glycosylation, mucin contains lots of sugar groups for attracting water, physically contributing to the gel formation of the PCL (Button et al., 2012) and forming viscoelastic mucus (Puchelle et al., 1995). Interestingly, MUC5B secretion is upregulated by airway inflammation (Kim et al., 2004a) and required for airway defense (Roy et al., 2014). Acting as the first line to remove invading bacteria, mucociliary clearance is defective in CF (Matsui et al., 1998; Hoegger et al., 2014; Esther et al., 2019; Keith et al., 2022), a major reason that facilitates bacterial colonization in CF airways.

Second, those bacteria that escape mucociliary clearance are targeted by a large number of antimicrobial proteins, such as lysozyme and lactoferrin and peptides, including β-defensins, LL-37 and CCL20 (Myszor and Gudmundsson, 2023) (Figure 1) which are secreted from surface and submucosal gland epithelia (Bals et al., 1998; Singh et al., 1998). These antimicrobials destroy bacteria mainly by perforating the bacterial outer membrane and cell wall (Parker and Prince, 2011; Andersson et al., 2016) to cause osmotic damage to the cell (Ellison and Giehl, 1991). For example, lysozyme kills Gram-positive bacteria by degrading the surface peptidoglycan wall (Wadstrom and Hisatsune, 1970), whereas lactoferrin by binding and lowering available ferrous ion inhibits the growth of Gram-negative bacteria (Ellison and Giehl, 1991). The antimicrobial peptide magainin 2 amide forms pores in the lipid membrane of bacteria, leading to cell lysis (Wenk and Seelig, 1998), whereas other antimicrobial peptides are bacteriostatic by penetrating into bacteria and interfering with cellular metabolism (Andersson et al., 2016), such as β-defensins (Sugiarto and Yu, 2007) and indolicidin (Ghosh et al., 2014) that bind DNA duplexes and then inhibit DNA synthesis (Subbalakshmi and Sitaram, 1998). Interestingly, after binding to the receptor on the cell membrane, β-defensins (Yang et al., 1999) and LL-37 (De et al., 2000; Niyonsaba et al., 2002) are also chemotactic for immune cells, including mast cells, monocytes and T cells (Andersson et al., 2016; Myszor and Gudmundsson, 2023). Finally, chemical compounds, such as H_2_O_2_ and SCN^−^, are secreted by airway epithelial cells to kill bacteria (Fischer, 2009; Ashtiwi et al., 2021).

Third, bacteria colonization on the surface of epithelia induces the cellular immune response (Figure 1), followed by tissue inflammation and damage that eventually cause lung failure in people with CF [for details, see the review (Ribeiro et al., 2023)]. CFTR is also expressed in immune cells, such as neutrophils (Painter et al., 2006; Zhou et al., 2013), monocytes (Sorio et al., 2011; Zhang et al., 2022) and macrophages (Di et al., 2006), but it remains unclear whether CFTR dysfunction disrupts the immune response of these cells and whether this significantly contributes to the development of CF lung disease.

**FIGURE 2:**
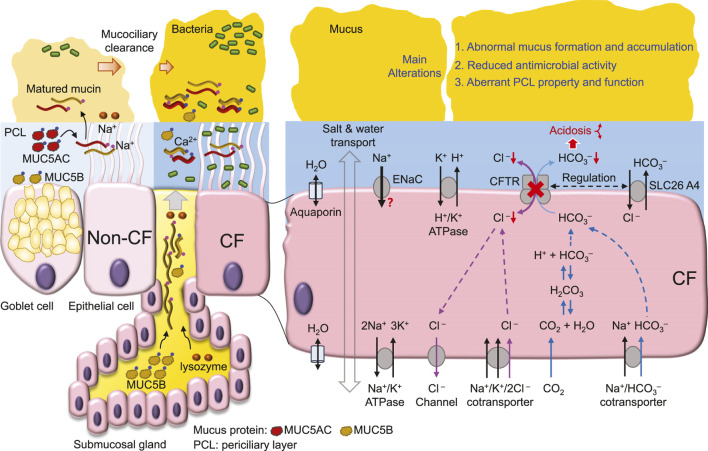
CFTR dysfunction-mediated CF airway abnormalities.

The following sections of this review focus on current research progress and perspectives about the mechanisms by which CFTR dysfunction causes defects in the 1st- and 2nd-levels of airway defense, mucociliary clearance and antimicrobial agents, respectively (Figure 2).

## 4 The Cl^−^ transport defect and the high salt hypothesis

CF-associated mutations diminish CFTR function primarily by reducing 1) mRNA synthesis, 2) protein expression, 3) channel regulation and 4) channel conductance, or by increasing 5) protein degradation at the cell membrane (Amaral and Farinha, 2013). For example, the most prevalent CF mutation ΔF508 by introducing intrinsic structural flaws (Chen et al., 2019) blocks CFTR protein expression at the cell membrane (Cheng et al., 1990), whereas ΔF508, G551D and G1349D mutations greatly decrease channel activity (Dalemans et al., 1991; Cai and Sheppard, 2002; Cai et al., 2006; Bompadre et al., 2007; Chen et al., 2009; Chen et al., 2017) with further alterations in channel responses to gating potentiators (Hwang et al., 1997; Cai and Sheppard, 2002; Cai et al., 2006; Bompadre et al., 2007) and intracellular pH (Chen et al., 2009; Chen et al., 2017). Therefore, the current drug therapy for CF aims to elevate the protein expression and channel activity of mutant CFTRs by correctors and potentiators, respectively (Bose et al., 2020).

Early studies (Sheppard et al., 1993; Sheppard et al., 1995) demonstrate that CF-associated mutations that cause more severe pancreatic insufficiency in people with CF display little or no CFTR-mediated transmembrane Cl^−^ transport. These data suggest that the deficit in transmembrane Cl^−^ transport is correlated with the level of CF epithelial disease. Consistent with this idea, in cultured CF human bronchial epithelia (HBE), bacterial killing on the apical surface is defective (Smith et al., 1996), possibly due to reduced bactericidal activity of lysozyme and lactoferrin caused by the high NaCl concentration in ASL (Smith et al., 1996). Although reduced Cl^−^ transport in CF epithelia may cause Cl^−^ (Smith et al., 1996; Matsui et al., 1998; Zabner et al., 1998; Kozlova et al., 2006) and Na^+^ (Zabner et al., 1998; Kozlova et al., 2006) retention in ASL, some studies find no difference in ASL Na^+^ (Matsui et al., 1998; Pezzulo et al., 2012) and Cl^−^ (Matsui et al., 1998) concentration between wild-type and CF epithelia. Of note, the reported ASL ion concentrations vary greatly among different studies, which employ distinct methods [e.g., (Verkman et al., 2003; Kozlova et al., 2006; Pezzulo et al., 2012), for details, see the review (Verkman et al., 2003)] to report higher (Smith et al., 1996) or unchanged (Matsui et al., 1998) concentrations in cultured CF epithelia compared to normal. Moreover, the sputum salt concentration is higher in people with CF than control subjects (Grandjean Lapierre et al., 2017). Therefore, a new and reliable method may be required to measure the salt concentration in ASL to resolve these controversial findings.

The high salt hypothesis is also challenged by evidence that aquaporin water channels are expressed on both the apical and basolateral membrane of airway epithelial cells (Kreda et al., 2001; Song et al., 2001). However, aquaporins have minor effects on airway humidification, ASL hydration and iso-osmolar fluid absorption (Song et al., 2001). Even if the osmolality is similar on either side of the apical membrane of airway epithelia, the salt concentration of ASL and cytosol may not be the same due to other factors such as extracellular mucins (Button et al., 2012; Henderson et al., 2014) and intracellular proteins that all contribute to the osmotic pressure affecting salt and water movement.

In addition, bacteria inoculation enhances epithelial fluid secretion in intact isolated swine tracheas (Luan et al., 2014). These data are consistent with the idea that CFTR-mediated Cl^−^ and fluid secretion may modulate the flow of mucus upon airways (Puchelle et al., 1995) to regulate mucociliary clearance of invading bacteria (Caverly et al., 2022). Thus, defective Cl^−^ transport of CF epithelia may impair bacterial eradication by mucociliary clearance, additionally to the high salt in ASL.

CFTR Cl^−^ transport is important for myeloperoxidase-mediated bactericidal hypochlorous acid (HOCl) production in the phagosome of neutrophils (Wang and Nauseef, 2022). Consequently, mutant CFTR may attenuate bacterial killing by neutrophils and cause immunodeficiency with abundant neutrophil inflammation in CF airways (Wang and Nauseef, 2022). CFTR also transports SCN^−^ (Linsdell, 2016). Compared to normal, the concentration of SCN^−^ in CF ASL is reduced in mice (Gould et al., 2010) and pigs (Lorentzen et al., 2011) but not humans (Lorentzen et al., 2011). Interestingly, the SCN^−^ concentration in ASL is about 30-fold higher than that in serum (Lorentzen et al., 2011), and people with CF showing higher ASL SCN^−^ levels display better lung function (Lorentzen et al., 2011). The data support the findings that SCN^−^ by reacting with H_2_O_2_ and HOCl acts as an antioxidant to neutralize peroxides and hence, protect epithelial cells from these oxidative injuries during neutrophil inflammation (Xu et al., 2009). Future work is required to assess the significance of peroxide-mediated injury in the pathogenesis of CF lung disease.

## 5 HCO_3_^−^ transport and the ASL acidosis hypothesis

HCO_3_^−^ conductance is about one-quarter that of Cl^−^ conductance for wild-type CFTR (Poulsen et al., 1994) (Figure 2). CF-associated mutations that diminish CFTR Cl^−^ transport likely also impair HCO_3_^−^ secretion (Smith and Welsh, 1992), whereas the conductivity ratio of HCO_3_^−^/Cl^−^ is somewhat variable among mutant CFTRs (Choi et al., 2001). Apical HCO_3_^−^ efflux can be enhanced by reciprocal interactions and stimulations between CFTR and other HCO_3_^−^ transporters, such as SLC26A3 (DRA) and SLC26A6 (Ko et al., 2002; Ko et al., 2004), whereas CF mutations may disrupt this cooperative HCO_3_^−^ secretion (Choi et al., 2001; Ko et al., 2002). These findings suggest an important role for CFTR in regulating HCO_3_^−^ secretion into ASL.

The importance of the HCO_3_^−^ deficit in CF is evident from the positive correlation of pancreatic sufficient CF patients with HCO_3_^−^ transport across the cell membrane (Choi et al., 2001). Moreover, HCO_3_^−^ deficit-induced ASL acidosis in *CFTR* ^*−/−*^ newborn pig airways results in impaired bacterial killing (Pezzulo et al., 2012; Shah et al., 2016a) partly due to reduced antimicrobial activity of lysozyme, lactoferrin, β-defensin-3 and LL-37 in the airways (Pezzulo et al., 2012; Abou Alaiwa et al., 2014b). In CF airways, the acidic pH of ASL increases ASL viscosity (Tang et al., 2016), causing abnormal mucus movement (Hoegger et al., 2014). These data emphasize the pathological development of CF airway disease with acidic ASL disrupting airway bacterial killing and mucus movement, resulting in chronic bacterial colonization (Stoltz et al., 2015) (Figure 2).

A direct test of this hypothesis is exploring whether CFTR dysfunction acidifies the ASL of people with CF and the apical liquid of cultured epithelia or cells. Previous studies (Pezzulo et al., 2012; Shah et al., 2016a; Li et al., 2016; Tang et al., 2016) indicate that the ASL pH of cultured CF pig airway epithelia is more acidic than that of non-CF. Using a pH microelectrode, other studies demonstrate that the apical pH of cultured epithelia or the extracellular pH of epithelial cells from different human cell lines, such as CFBE41o− (Simonin et al., 2019), Calu-3 (Shan et al., 2012), IB3-1 vs. C38 (Valdivieso et al., 2019) and CuFi-1 vs. NuLi cells (Muraglia et al., 2019) or from human bronchial epithelial cells (Coakley et al., 2003; Haggie et al., 2016; Scudieri et al., 2018; Simonin et al., 2019; Gianotti et al., 2020) is consistently more acidic in CF than that in non-CF. The pH difference (∆pH) between CF and non-CF among these studies is about 0.2–0.65 (Coakley et al., 2003; Pezzulo et al., 2012; Shan et al., 2012; Haggie et al., 2016; Scudieri et al., 2018; Simonin et al., 2019; Gianotti et al., 2020), whereas well-differentiated epithelia with better epithelial polarization and more CFTR expressed in the apical membrane than those of less differentiated epithelia (Sheppard et al., 1994), exhibit larger ∆pH (∼0.5) decreases in CF than non-CF epithelia (Scudieri et al., 2018; Gianotti et al., 2020). These data are consistent with the idea that the loss of CFTR-mediated HCO_3_^−^ secretion in CF airways leads to ASL acidosis (Pezzulo et al., 2012; Stoltz et al., 2015). Moreover, transgenic mice overexpressing the proton pump, non-gastric H^+^/K^+^ ATPase (ATP12A) in the apical membrane of airway epithelia have reduced ASL pH and develop CF-like lung disease (Shah et al., 2016b), further supporting this ASL acidosis hypothesis.

However, direct measurements of ASL pH in animals or human subjects with or without CF reveal complex mechanisms that participate in ASL pH regulation. When compared to that of wild-type animals, the ASL pH is more acidic in CF newborn pigs (Pezzulo et al., 2012; Shah et al., 2016b) and 1–6 month-old rats (Birket et al., 2018) but not in adult mice (Jayaraman et al., 2001). In humans, CF neonates (<1 month old) exhibit ASL pH lower than healthy controls (Abou Alaiwa et al., 2014a; Abou Alaiwa et al., 2018) and CF submucosal gland secretion from nasal biopsies is also more acidic than that from non-CF (Song et al., 2006). However, no difference in ASL pH between CF and non-CF is found in either children (McShane et al., 2003; Abou Alaiwa et al., 2014a; Schultz et al., 2017; Abou Alaiwa et al., 2018) or adults (McShane et al., 2003). A study using primary cultures of human airway epithelial cells observed no difference in the pH of the apical fluid between CF and non-CF (Schultz et al., 2017). Conversely, with HCO_3_^−^-free culture medium, cultured CF epithelia still show ASL pH lower than non-CF (Scudieri et al., 2018). Thus, in addition to CFTR-mediated HCO_3_^−^ secretion, age-associated adaptions (Abou Alaiwa et al., 2018) and other regulatory mechanisms, such as ATP/histamine-stimulated proton secretion (Song et al., 2006), pendrin (SLC26A4, a Cl^−^/anion exchanger) (Haggie et al., 2016; Simonin et al., 2019) (Figure 2) and H^+^/K^+^-ATPase (ATP12A) (Scudieri et al., 2018; Simonin et al., 2019) may all contribute to ASL pH regulation.

For instance, pendrin containing 870 amino acids is an electroneutral transporter exchanging Cl^−^ for HCO_3_^−^, I^−^, NO_3_^−^, SCN^−^ or HCO_2_^−^ across the cell membrane (Haggie et al., 2016; Tamma and Dossena, 2022). In airway epithelia, pendrin is found abundantly on the apical membrane of ciliated epithelial cells but little in submucosal glands (Kim et al., 2019) and its gene expression is not significantly increased in CFTR-rich ionocytes (Rehman et al., 2020). Cl^−^/HCO_3_^−^ exchange by pendrin in airway epithelial cells is upregulated by the cytokines IL-13 to reduce ASL depth (Haggie et al., 2016), by IL-17A (Adams et al., 2014), by IL-17 together with TNF-α to alkalinize ASL pH (Rehman et al., 2020) and by IL-4 to stimulate pendrin-mediated HCO_3_^−^ secretion and CFTR-mediated electrogenic Cl^−^ efflux (Kim et al., 2019). These data suggest that pendrin promotes Cl^−^/HCO_3_^−^ transport during inflammation (Haggie et al., 2016; Kim et al., 2019; Rehman et al., 2020), but whether functional interplay between CFTR and pendrin (Rehman et al., 2020; Tamma and Dossena, 2022) is defective in CF airway leading to loss of HCO_3_^−^ secretion remains to be explored in future work.

As most studies demonstrate that the apical solution of cultured epithelia is more acidic in CF than non-CF, the data suggest that CF epithelia have defective ASL pH regulation. Although CFTR-mediated HCO_3_^−^ secretion is defective in CF epithelia (Smith and Welsh, 1992; Choi et al., 2001) and plays a role in ASL acidosis of neonates with CF (Abou Alaiwa et al., 2014a; Abou Alaiwa et al., 2018), it remains unclear whether abnormalities resulting from ASL acidosis in CF neonates would be the major factor causing later progressive deteriorations in the lung function of children and adult patients. Future investigation of the relationship between ASL pH, salt concentration, antimicrobial activity and the physicochemical properties of mucus may reveal the major underlying mechanism of CF lung disease caused by defective CFTR ion transport.

## 6 The role of epithelial Na^+^ transport in bacterial eradication in CF airways

Comprehensive reviews about the Na^+^ hyperabsorption or low volume hypothesis can be found in previous literatures (Tarran et al., 2006; Lazarowski and Boucher, 2021). The main concept of this hypothesis is that CFTR dysfunction in CF may result in hyperactivity of ENaC (Stutts et al., 1995; Matsui et al., 1998), leading to transepithelial hyperabsorption of NaCl and water (Matsui et al., 1998). Consequently, the height of ASL is reduced, causing cilia bending and deformation, impairing mucociliary clearance (Matsui et al., 1998) and preventing bacterial eradication (Tarran et al., 2006). This hypothesis is also supported by transgenic mice overexpressing the ENaC-β subunit in airway epithelia, which generates CF-like lung inflammation and disease (Mall et al., 2004).

Two ideas to explain CFTR-mediated regulation of ENaC activity are the modulation of channel activity by the intracellular Cl^−^ concentration and direct interactions between the two channels (Berdiev et al., 2009). However, the basis of the low volume hypothesis, including ENaC hyperactivity by loss of CFTR function and Na^+^ hyperabsorption in CF epithelia is not supported by several experimental approaches, such as patch-clamp studies of ENaC channel activity (Nagel et al., 2005), the measurement of transepithelial Na^+^ absorption (Chen et al., 2010; Itani et al., 2011; Willumsen and Boucher, 1991a, b) and amiloride-sensitive changes in short-circuit currents of cultured pig airway epithelia (Chen et al., 2010). Similarly, treatment of CF airway epithelia with hypertonic solution that likely facilitates epithelial water secretion against Na^+^ and fluid hyperabsorption achieves only a moderate, short-term improvement of lung function in people with CF (Wark and McDonald, 2018). Therefore, deeper insight into the interaction of CFTR and ENaC is required to further investigate this hypothesis.

## 7 CFTR-mediated regulation of membrane transporters and intracellular proteins

CFTR interacts with many membrane transporters to regulate epithelial function (Iazzi et al., 2023). For example, CFTR modulates the activity of the Na^+^/H^+^ exchanger (NHE) (Ahn et al., 2001) and Cl^−^/HCO_3_^−^ exchanger (Lee et al., 1999; Ko et al., 2004) to adjust extra- and intracellular pH, implicating the function of these transporters in ASL pH regulation. CFTR through its N-terminal peptide interacts with filamin A (Smith et al., 2010) and the SNARE proteins syntaxin 1A (Naren et al., 1997) and SNAP-23 (Cormet-Boyaka et al., 2002). Moreover, CFTR uses the type I PDZ-binding motif (D/E)T(R/K)L in its C-terminal peptide to interact with Na^+^/H^+^ exchange regulatory cofactor 1 (NHERF1) (Hall et al., 1998; Martin et al., 2020), NHERF2 (Sun et al., 2000), NHERF3 (also known as PDZK1, CAP70 or NaPi-Cap1) (Wang et al., 2000), NHERF4 (IKEPP) (Hegedus et al., 2003), CFTR-associated ligand (CAL) (Cheng et al., 2002) and Shank2 (Kim et al., 2004b). Finally, the R domain of CFTR interacts with the antisigma factor antagonist (STAS) domain of SLC26 transporters (Ko et al., 2004), calmodulin (Bozoky et al., 2017) and other intracellular proteins (Bozoky et al., 2013). However, it remains unclear whether defects in these CFTR-mediated interactions or regulation play a significant role in CF lung pathogenesis.

## 8 The association of CFTR dysfunction with mucus accumulation in CF

A major hallmark of CF disease is the accumulation of mucus in the lumen of different ducts, such as the airways, gastrointestinal tract, bile duct and reproductive systems (Hansson, 2019). In CF airways, thick and sticky mucus detain airborne bacteria, resulting in bacterial colonization, biofilm formation and later bacterial invasion of the epithelium, inducing severe and recurrent inflammation (Reece et al., 2021; Shteinberg et al., 2021). Therefore, the removal of airway surface mucus facilitates bacterial eradication in CF (Belli et al., 2021).

Airway mucus is secreted by Goblet cells in the surface epithelium and submucosal glands (Morrison et al., 2019) and is matured in ASL after transformation from a core structure to a hydrated and extended mucin molecule (Abdullah et al., 2017) (Figure 1). In this maturation process, Ca^2+^ in mucin bundles is replaced by Na^+^ in ASL, followed by mucin hydration and extension (Verdugo, 1990; Kesimer et al., 2010; Morrison et al., 2019) (Figure 1). In CF airways (Figure 2), mucus secretion is exaggerated due to bacterial contact with the epithelium (Ben Mohamed et al., 2012), cytokine secretion, such as IL-1 (Balazs and Mall, 2019), IL-8 (Ben Mohamed et al., 2012), and IL-13 (Zhu et al., 1999), and at a later stage, the hyperplasia of Goblet cells and glands (Morrison et al., 2019). In addition, other factors such as ASL acidosis (Ambort et al., 2012; Tang et al., 2016), reduced HCO_3_^−^ secretion (Saint-Criq et al., 2022) and airway surface dehydration (Abdullah et al., 2017) may alter the viscosity (Tang et al., 2016), maturation (Abdullah et al., 2017) and movement (Matsui et al., 1998; Hoegger et al., 2014) of mucus, likely impairing the mucociliary clearance mechanism (Figure 2). Thus, the dehydration (Abdullah et al., 2017) and deformation (Tang et al., 2016) of mucus that impairs bacterial eradication have been the focus of much recent attention in CF airway research.

CFTR dysfunction facilitates airway bacterial colonization and infection, leading to excess inflammation (Ribeiro et al., 2023) that further causes tissue remodeling and damage, such as epithelial-mesenchymal transition (Quaresma et al., 2022). These epithelial disorders may form a positive but detrimental feedback loop, where inflammation-induced Goblet cell hyperplasia (Morrison et al., 2019) continuously stimulates mucus secretion and then mucus accumulation in CF airways further traps more bacteria, hyperactivating the inflammatory responses of epithelia. In addition, sterile inflammation triggered by mucus plugging via the interleukin-1 signaling pathway (Balazs and Mall, 2019) amplifies this vicious cycle. Indeed, an urgent task in future research is to effectively interrupt this cycle to develop important new treatments for CF lung disease.

## 9 Closing remarks

Over 3 decades of CF research has demonstrated that lack of CFTR-mediated HCO_3_^−^ secretion and subsequent ASL acidosis provides a convincing mechanism that leads to abnormal mucus and bacterial eradication (Stoltz et al., 2015) at least in the CF airways of neonates (Abou Alaiwa et al., 2014a; Abou Alaiwa et al., 2018). Deficits of CFTR-mediated Cl^−^ transport and regulation of other proteins may also contribute to the pathogenesis of CF. Clarifying the role of each CFTR functional defect in the development of CF lung disease may reveal the major routes responsible for the cycles of bacterial infection, inflammation and mucus accumulation in CF airways (Ribeiro et al., 2023). New treatments that prohibit airway bacterial colonization or abnormal mucus accumulation would be promising approaches to treat CF lung disease and perhaps also other infectious lung diseases.
